# Impact of the COVID-19 pandemic on *Bordetella pertussis* infections in England

**DOI:** 10.1186/s12889-022-12830-9

**Published:** 2022-02-28

**Authors:** Elise Tessier, Helen Campbell, Sonia Ribeiro, Yuma Rai, Simon Burton, Partho Roy, Norman K. Fry, David Litt, Gayatri Amirthalingam

**Affiliations:** 1 Immunisations and Countermeasures Division, UK Health Security Agency, 61 Colindale Avenue, NW9 5EQ London, England; 2 Respiratory and Vaccine Preventable Bacteria Reference Unit, UK Health Security Agency, 61 Colindale Avenue, NW9 5EQ London, England

## Abstract

**Background:**

In March 2020, England went into its first lockdown in response to the COVID-19 pandemic. Restrictions eased temporarily, followed by second and third waves in October 2020 and January 2021. Recent data showed that the COVID-19 pandemic resulted in reduced transmission of some invasive diseases. We assess the impact of the COVID-19 pandemic on pertussis incidence and on the immunisation programme in England.

**Methods:**

We assessed trends in pertussis cases from 2012 to 2020 by age group and month. Incidence from the time that England eased its initial lockdown measures in July 2020 through to summer 2021 was calculated and the incidence rate ratios of pertussis cases from five years prior to the pandemic (July 2014 – June 2019) compared to the same time period during the pandemic (July 2020 – June 2021). Vaccine coverage estimates for pertussis containing vaccines were reviewed for the maternal and childhood programmes.

**Results:**

A substantial decline in pertussis cases was observed from April 2020 onwards, marking the lowest number of cases in the last decade. Pertussis incidence dropped in all age groups, particularly among infants less than one year old (0.50 / 100,000 during July 2020 to June 2021 compared to 24.49/ 100,000 from July 2014 to June 2019). The incidence rate ratio was 0.02 (95% CI 0.01 to 0.02) for July 2014 to June 2019 (pre-pandemic) compared to the pandemic period of July 2020 to June 2021. None of the cases had a co-infection with SARS-CoV-2. Vaccine coverage for infants born between January to March 2020 with three doses of pertussis vaccine by 12 months of age decreased by 1.1% points compared to infants born between January to March 2019 (91.6% and 92.7%, respectively). Prenatal pertussis coverage for the 2020 to 2021 financial year was 2.7% points lower than the year prior to the pandemic (70.5% and 76.8%, respectively).

**Conclusions:**

Lockdown measures due to the COVID-19 pandemic have had a significant impact on pertussis transmission. With the easing of restrictions it is important to continue monitoring pertussis cases in England alongside coverage of the maternal and childhood immunisation programmes.

## Background

*Bordetella pertussis* (pertussis) is a vaccine-preventable respiratory infection caused by the bacterium *Bordetella pertussis*. The World Health Organization (WHO) reported over 151,000 cases of pertussis in 2018, with the highest burden of disease among infants [1, 2].

In October 2004, a less reactogenic acellular pertussis vaccine was introduced in the United Kingdom (UK) routine infant schedule, replacing the whole cell pertussis vaccine offered at 8, 12 and 16 weeks [3, 4]. An acellular pertussis pre-school booster vaccine was introduced in 2001 and is offered three years after completion of the primary course at 3 years 4 months. In 2012 the UK experienced a pertussis outbreak with over 10 times the number of confirmed cases compared to previous peak years and the highest disease incidence in infants < 3 months of age. Results from mathematical modelling indicate that whole cell pertussis vaccines induce a longer duration and improved protection against pertussis compared to acellular vaccines, therefore the change to acellular vaccine is thought to have contributed to increased levels or pertussis with predictions that levels of transmission will persist at heightened levels in the future [5]. In response to the outbreak, an emergency maternal vaccination programme was implemented to protect young infants before they became eligible for their first vaccine at 8 weeks through transplacental transfer of maternal antibodies. The estimated effectiveness of maternal vaccination against infant disease is high and since the implementation of the programme, pertussis case numbers have fallen overall, with no further national outbreaks observed [6, 7]. From 2019, the delivery of maternal pertussis vaccinations has become a routine immunisation programme [8].

In England, a cyclical pattern of pertussis cases is observed every 3–4 years with an annual peak occurring during the months of July to September. A cyclical peak in pertussis cases was expected in the 2020 calendar year after increasing case numbers were observed during the later months of 2019. However, in March 2020, the UK went into its first COVID-19 lock-down where physical distancing measures were implemented which included school closures, the prohibition of gatherings and non-essential use public transportation and advise to work from home [9]. Since March 2020, measures including social distancing and compulsory mask wearing continued until 18 July 2021 and the UK has entered a series of local tiered restrictions and nationwide lockdowns throughout the remainder of 2020 and up to July 2021.

Recent data showed that after COVID-19 pandemic control measures were introduced there was reduced transmission of invasive disease due to *Streptococcus pneumoniae, Haemophilus influenzae, Neisseria meningitidis* globally and for influenza in England [10–13].

Like pertussis, SARS-CoV-2 (the virus causing COVID-19 disease) is transmitted via respiratory droplets. SARS-CoV-2 spreads from an infected individual during either the symptomatic or pre-symptomatic phase of infection and has an incubation period of 2 to 14 days [14]. SARS-CoV-2 infection can lead to severe lung injury and can affect other organs and cause systemic inflammation leading to symptoms affecting gastrointestinal, cardiovascular, haematological, renal, musculoskeletal and endocrine systems [14]. SARS-CoV-2 impacts all ages, though unlike pertussis, the vast majority of paediatric cases are mild [15]. SARS-CoV-2 has rapidly spread across the globe and has a basic reproduction number (R_0_) ranges from 2 to 2.5 [14]. Pertussis however is far more infectious with R_0_ estimates based on a next generation matrix of 5.5 across five countries including England and Wales [16] .

By 30 June 2021, there were 4,853,425 people in the UK who had tested positive for SARS-CoV-2 either by laboratory-confirmed PCR test in the UK and/ or by a rapid lateral flow test, in England only [17].

The aim of our study was to summarise and assess the impact of measures introduced to control COVID-19 on pertussis incidence and vaccine coverage in England.

## Methods

### Cases, incidence and positivity of pertussis

The UK Health Security Agency (UKHSA) is responsible for the national surveillance of vaccine-preventable infections with detailed individual follow-up of all laboratory-confirmed pertussis cases in England. Laboratory confirmation occurs at the local hospital microbiology laboratory (culture); through the UKHSA Specialist Microbiology Service laboratory network (PCR available through the regional network since July 2014 for all age groups, previously only offered for hospitalized infants by the national reference laboratory); or at the national reference laboratory (serological testing available since 2001 for all age groups and, since January 2013, oral fluid antibody testing for suspected cases initially aged 8–16 years, extended to those aged 5–16 years from July 2013, and to 2–16 years from May 2018). Both serology and oral fluid testing are based on demonstrating significant anti-PT immunoglobulin G titres above a predefined threshold considered indicative of recent infection (with the caveat that results may be confounded by pertussis vaccination within the previous year). All laboratory-confirmed cases are followed up with the patients’ general practitioner (GP) to collect clinical and epidemiological data including vaccination history and, for infants born after 1 October 2012, maternal vaccination status [6, 18]. Quarterly and annual reports summarising confirmed pertussis cases in England are regularly published [19]. We assessed the trends in the number of pertussis cases from 2012 to 2020 by age group and month. To assess the impact of the pandemic on pertussis incidence from when England eased its initial lockdown measures we selected a date of July 2020 to exclude any surplus in cases that were infected before the lockdown due to the delay in the development of symptomatic disease and in seeking medical advice. We calculated the incidence rate ratios (IRR) of pertussis cases by age (< 1 year; 1–4 years; 5–14 year; 15 years and above) from the months July to June each year for the five years prior to the COVID-19 pandemic (July 2014 – June 2019) compared to the same time period during the pandemic (July 2020 – June 2021).

Finally, to assess whether the number of pertussis cases was impacted by testing, the percent positivity of serology, PCR and oral fluid tests was calculated from January 2014 to June 2021 by dividing the total number of positive cases by the total number of oral fluid, PCR and serological pertussis tests reported to UKHSA each month. Culture negative samples were not included in the percent positivity analysis, but overall culture samples accounted for a small proportion of positive tests 573/26,808 (2.1%).

### Vaccine coverage

UKHSA is responsible for monitoring and evaluating routine immunisation programmes, including vaccine coverage for all pertussis containing vaccines [20]. We summarised vaccine coverage by financial year (April to March) from three sources.

- Prenatal pertussis vaccine coverage among women who have delivered and were vaccinated prior to delivering. Data were collected from GP records from ImmForm [21].
- Completed course of DTaP/IPV/Hib/HepB (Hexavalent vaccine) vaccine evaluated at 12 months from the COVER collection, which evaluates coverage from the Child Health Information Systems (CHIS) across England [22, 23].
- Dose 3 coverage of Hexavalent vaccine evaluated at 6 months from ImmForm [21], which evaluates vaccine coverage from GP practices records across England.

### Pertussis and SARS-CoV2 infections

All pertussis cases from July 2020 to June 2021, were linked to all SARS-CoV-2 positive test results from Pillar 1 and 2 (hospitalised, hospital workers and community respectively) from the official line list on 10 August 2021. Cases were linked using National Health Service (NHS) number, name of the individual and date of birth. Individuals who tested positive for SARS-CoV-2 and had onset of confirmed pertussis within a 28-day period were considered to have a co-infection. When the pertussis symptom onset date was not available, date of sample was used.

## Results

### Cases, incidence and positivity of pertussis

**Fig. 1:**
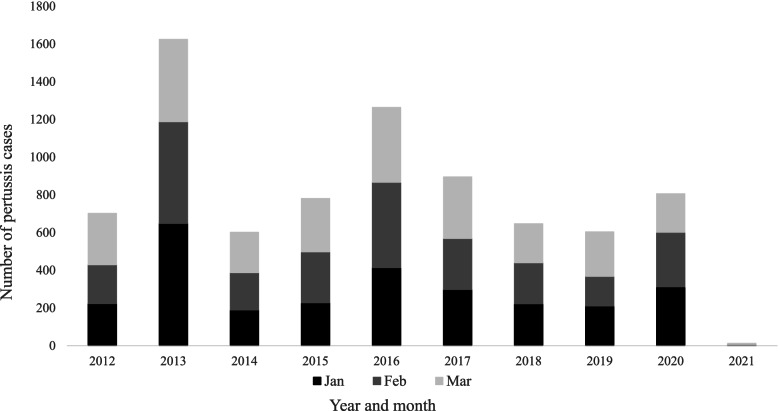
Total number of pertussis cases between January and March annually, England 2012 to 2021

The overall number of laboratory-confirmed cases of pertussis cases drastically dropped after the outbreak in 2012/2013, with an expected cyclical increase in 2016/2017 and the beginning of a peak observed again in 2019/20. Cases in 2019 remained consistently higher from March to December, than during the same months in 2018. The number of cases remained high with 311, 290 and 205 cases in January, February and March 2020, respectively, compared to 209, 158 and 237 cases in January to March 2019, consistent with the possibility of 2020 becoming a peak year (Fig. 1).

**Fig. 2:**
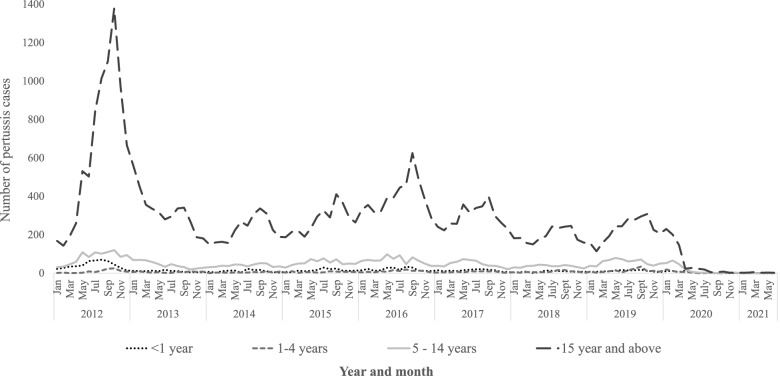
Total number of pertussis cases from January 2012 to June 2021 by month and age group

However, from April 2020 onwards the number of pertussis cases rapidly dropped and case numbers have remained lowest in the last decade with a total of 209 cases from April 2020 to June 2021 (Fig. 2).

Among all age groups, social distancing and precautionary measures implemented due to the COVID-19 pandemic were protective against pertussis with an overall 98% (IRR 0.02, ( 95% CI 0.01 to 0.02)) reduction in the overall rate of pertussis cases from July 2020 to June 2021 compared to the same annual periods from 2014 to 2019 (Table 1). Between July 2020 and June 2021, the incidence rate ratios ranged from 0.01 (95% CI 0.01–0.02) among 5-14-year olds to 0.06 (95% CI 0.02 to 0.15) among 1-4-year olds (Table 1).

**Table 1:** Number of *Bordetella pertussis* cases, population estimates, incidence per 100,000 and incidence rate ratio (IRR) for cases in July 2020 to June 2021 compared to cases during the same months for 2014 to 2019

Age group	Average population estimate from 2014–2019	Average Number of cases from July 2014 to June 2019	Incidence per 100,000	Population estimate 2020	Number of cases from July 2020- June 2021	Incidence per 100,000	IRR (95% CI)
< 1 year old	657,513	161	24.49	601,913	3	0.50	0.02 (0.00 to 0.06)
1–4 years old	2,747,754	86	3.13	2,637,534	5	0.19	0.06 (0.02 to 0.15)
5–14 years old	6,512,625	613	9.41	6,975,037	8	0.11	0.01 (0.01 to 0.02)
15 years and above	45,275,632	3,335	7.37	46,335,654	60	0.13	0.02 (0.01 to 0.02)
Total	55,193,524	4,195	7.60	56,550,138	76	0.13	0.02 (0.01 to 0.02)

**Fig. 3:**
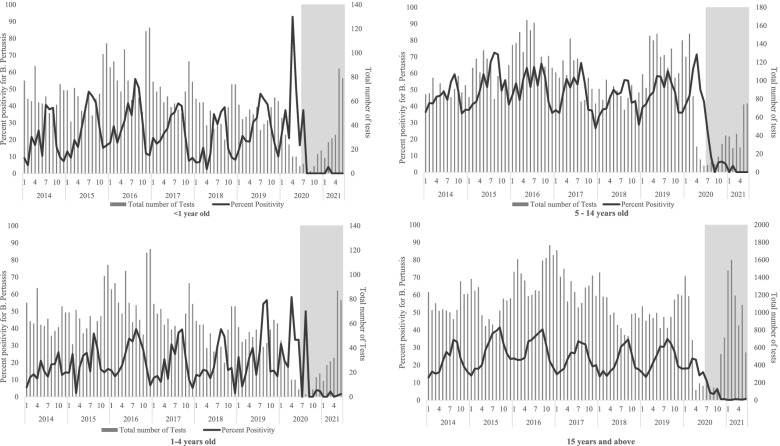
Total number of oral fluid, PCR and serology *B. Pertussis* tests and percent positivity by age group from January 2014 to June 2021 in England. The section in grey represents when SARS-CoV-2 restrictions changed across England.* *Grey area represents when lockdown measures changed in England

Finally, the total number of laboratory tests for pertussis (PCR, serology and oral fluid tests combined) and their percent positivity dropped among all age groups from July-August 2020 onwards to the lowest positivity rates observed since before 2014. However, an increasing number of tests was observed, particularly among those under 5 and in those aged 15 years and above, from September 2020 onwards, although the percent positivity remained very low (Fig. 3).

### Vaccine coverage

**Fig. 4:**
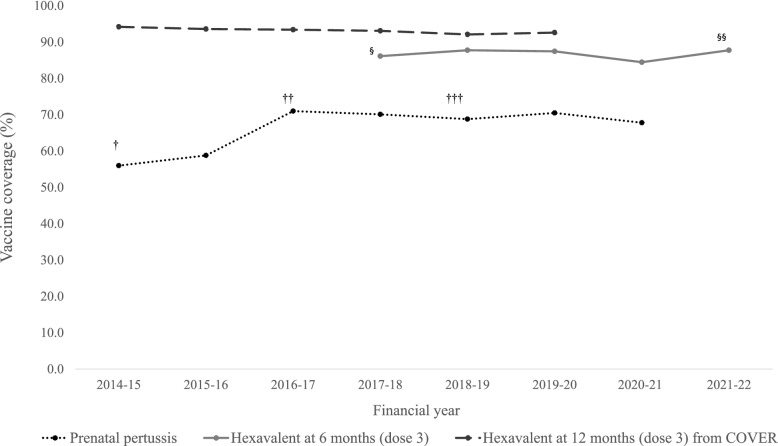
Vaccine coverage for the prenatal pertussis vaccination programme, completed course of Hexavalent vaccine observed at 6 months from ImmForm and completed course of Hexavalent vaccine at 12 months from the COVER collection. † Prenatal pertussis vaccines coverage among pregnant women vaccinated up to 14 weeks prior to delivery. † † Prenatal pertussis vaccines coverage among pregnant women vaccinated up to 16 weeks prior to delivery and excluding miscarriages and stillbirths regardless of gestational age. † † † Prenatal pertussis vaccines coverage among pregnant women vaccinated up to 26 weeks prior to delivery and excluding miscarriages and stillbirths regardless of gestational age. § Hexavalent vaccine coverage for 2014/15 estimates based on monthly extracts from June 2017 to March 2018. §§ Hexavalent vaccine coverage for 2021/22 estimates based on monthly extracts from April 2021 to June 2021

The prenatal pertussis vaccination programme introduced initially as an emergency programme as a result of the large outbreak in 2012 has remained consistently around 68 to 71% since 2016/17 when the denominator or eligible women was amended (see caveats in Fig. 4). Annual prenatal pertussis coverage for the 2020 to 2021 financial year was 2.7% points lower than during the 2019 to 2020 financial year (prior to the pandemic), dropping from 76.8% compared to 70.5%. Vaccine coverage in 2019/20 was the lowest since the change in the denominator in 2016/17 (Fig. 4).

Vaccine coverage from the COVER collection indicates a decreasing trend in coverage from 2015/14 to 2018/19 from 94.2 to 92.6%. Vaccine coverage from the 2019/20 financial year (April 2019 to March 2020) showed the first upward trend in coverage in the five years (Fig. 4), though this data reflects only those turning 12 months, 24 months and 5 years old up to March 2020.

For more timely estimates of the impact of COVID-19 on pertussis containing vaccines in infants, ImmForm data for infants turning 6 months old showed a decrease in completed Hexavalent vaccine coverage by 3.0% points in 2020/21 compared to 2019/20. An increasing trend in 2021/22 has been observed though this must be viewed with caution as the data only consist of data from April 2021 to June 2021 (see caveats in Fig. 4).

### Pertussis and SARS-CoV2 infections

Of the 76 pertussis cases between July 2020 to June 2021, a total of 8 (10.5%) subsequently had SARS-CoV-2. No individuals had both infections within 28 days, therefore there were no SARS-CoV-2 – pertussis co-infections. Furthermore, there were no individuals with pertussis succeeding a SARS-CoV-2 infection.

## Discussion

Based on epidemiological observations of pertussis trends in England, an anticipated peak year was expected in 2020. However, with the first national COVID-19 lockdown implemented in England from 23 to 2020, the number of pertussis cases in all age groups declined sharply, from April onwards.

When evaluating the incidence and incidence rate ratios from June 2020 to July 2021 compared to the same time periods in the previous five years, there was a significant drop temporally associated with the COVID-19 pandemic. Our findings concur with observations of pertussis cases in France where researchers observed an overall decrease in adjusted incidence rate ratios of 0.102 (95% CI 0.040–0.256) in outpatient laboratories and 0.216 (95% CI 0.071–0.656) in 41 paediatric hospitals from 2013 to 2020 across France due to the COVID-19 pandemic [24].

The sustained low numbers of pertussis cases are likely to be a result of measures to prevent the spread of COVID-19. The highest rates of pertussis are typically seen in infants and children. It is possible that school and nursery closures, social distancing and mask-wearing policies caused the large reduction in cases among children and infants and subsequent infection in adults caring for their children. A similar reduction has been observed throughout the pandemic for invasive *S. pneumoniae* cases in 26 countries and territories [10]. However, it is possible that other, unmeasurable factors also lowered the rate of pertussis. For example, individuals may have thought they had COVID-19, which has similar symptoms to pertussis. Such individuals may have followed government advice to stay at home or have been tested for SARS-CoV-2 instead of pertussis. It is uncertain all people who tested negative for SARS-CoV-2 would have subsequently been tested for other respiratory diseases, such as pertussis. However, our results indicate that pertussis testing resumed with the easing of the restrictions. Testing was higher among the older age groups in particular, which is most likely attributable to multiplex PCR testing available in hospital settings, which were developed to detect several respiratory pathogens including SARS-CoV-2 and pertussis [25]. It is of utmost importance to continue microbiological surveillance of pertussis disease, particularly among those with a negative SARS-CoV-2 test, and closely monitor across the population as social distancing for COVID-19 relaxes.

We found a concurrent drop in pertussis-containing vaccination rates during the COVID-19 pandemic. A drop in prenatal pertussis vaccination among pregnant women will result in an increased number of infants susceptible to pertussis in the first 8 weeks of life, prior to being eligible for their first dose of hexavalent vaccine [7]. Coverage for children born between January to March 2020 (first eligible for vaccines during the first lockdown) with three doses of pertussis vaccine by 12 months of age decreased by 1.1% points compared to infants born between January and March 2019 [22, 26]. Drops in coverage among pregnant women and infants risks increased transmission of pertussis disease as social distancing measures relax, with infants and young children that are not fully vaccinated more vulnerable for severe disease. Furthermore, drops in coverage can result in outbreaks, as seen in primary and secondary schools in England in the recent years [27, 28].

## Conclusions

In England, pertussis incidence has dramatically declined following the introduction of measures to control the COVID-19 pandemic. These results concur with findings for other diseases in England and globally, even for diseases where viral infections are known to increase susceptibility to bacterial infections such as meningococcal disease and pneumonia [10, 13].

England removed social distancing measures on 19 July 2021 for the first time since late March 2020 with individuals now allowed to visit in large groups indoors and no longer required to wear a mask, though people are encouraged to continue wearing them in enclosed and busy spaces. Subsequent to the COVID-19 lockdowns, an increase in pertussis cases from summer 2021 has yet to be observed, though could occur. COVID-19 has placed a noteworthy impact on the transmission of other infectious diseases. For example, a shift in the seasonal pattern of paediatric respiratory syncytial virus (RSV) has been observed in both the northern and southern hemisphere thus making it difficult to forecast disease patterns and when to expect high disease burden [29]. RSV/pertussis co-infections in infants have been known to be observed during peak RSV season (September to October) in the northern hemisphere, which overlaps with the seasonal trend for pertussis [30–32]. Despite raised cases of RSV following COVID-19 restrictions, we have not observed any signs of increased RSV/pertussis co-infections.

There have been neither SARS-CoV-2/ pertussis co-infections nor cases of individuals with a SARS-CoV-2 infection followed by pertussis observed in England to date. Whilst there may be less testing for pertussis following a positive SARS-CoV-2 test result, these findings suggest that SARS-CoV-2 infection does not increase susceptibility to pertussis and vice versa. Testing for both pertussis and SARS-CoV-2 should be conducted where symptoms may be suggestive of either disease.

Sustained high levels of vaccine coverage are known to reduce pertussis incidence [33, 34]. With decreases in vaccine coverage for pregnant women, infants and children, the proportion of susceptible individuals will increase in the absence of circulating disease. The increased proportion of young infants who are unimmunised or partially immunised leads to higher risk for severe complications. Additionally, delays in diagnosing pertussis in adolescents and adults (who often have atypical and mild symptoms and are thus drivers of pertussis infection [35, 36]. Finally, a year of social distancing measures indicates less exposure and natural boosting in adolescents and adults, thus increasing the risk of susceptibility for pertussis disease. The National Health Service in England is faced with rolling out the COVID-19 vaccination programme and the largest influenza vaccination programme in British history while general practitioners and local immunisation teams are faced with catching-up on routine immunisations to those who have been impacted by the pandemic such as MMR in children, HPV in adolescents and shingles for the elderly [22, 37–40].

The COVID-19 pandemic has had a marked impact on pertussis transmission and led to a decline in vaccine coverage for pertussis containing vaccines. As mixing patterns increase, pertussis is likely to re-emerge; however the overlap of cough symptoms in both COVID and pertussis risks pertussis cases being undiagnosed. This highlights the importance of continued vigilance. The continuation of microbiological surveillance and monitoring vaccine coverage in the upcoming months will be critical in assessing the extent of spread of pertussis disease and detecting any new emerging trends in these challenging times.

## Data Availability

Data may be available upon request from UKHSA.

## Abbreviations

Pertussis: Bordetella pertussis
COVID-19: Coronavirus disease
SARS-CoV-2: Severe-acute respiratory syndrome coronavirus 2 virus
surveillance: ongoing systematic collection and analysis of routinely collected data
vaccines: substance that is introduced in a persons body to create antibidies and prevent disease
